# Evolution of Archaellum Rotation Involved Invention of a Stator Complex by Duplicating and Modifying a Core Component

**DOI:** 10.3389/fmicb.2021.773386

**Published:** 2021-11-29

**Authors:** Trishant R. Umrekar, Yvonne B. Winterborn, Shamphavi Sivabalasarma, Julian Brantl, Sonja-Verena Albers, Morgan Beeby

**Affiliations:** ^1^Department of Life Sciences, Imperial College London, London, United Kingdom; ^2^Molecular Biology of Archaea, Faculty of Biology, Institute of Biology II, University of Freiburg, Freiburg, Germany; ^3^Spemann Graduate School of Biology and Medicine, University of Freiburg, Freiburg, Germany

**Keywords:** archaellar motor, stator complex, single particle analysis, molecular evolution, exaptation

## Abstract

Novelty in biology can arise from opportunistic repurposing of nascent characteristics of existing features. Understanding how this process happens at the molecular scale, however, suffers from a lack of case studies. The evolutionary emergence of rotary motors is a particularly clear example of evolution of a new function. The simplest of rotary motors is the archaellum, a molecular motor that spins a helical propeller for archaeal motility analogous to the bacterial flagellum. Curiously, emergence of archaellar rotation may have pivoted on the simple duplication and repurposing of a pre-existing component to produce a stator complex that anchors to the cell superstructure to enable productive rotation of the rotor component. This putative stator complex is composed of ArlF and ArlG, gene duplications of the filament component ArlB, providing an opportunity to study how gene duplication and neofunctionalization contributed to the radical innovation of rotary function. Toward understanding how this happened, we used electron cryomicroscopy to determine the structure of isolated ArlG filaments, the major component of the stator complex. Using a hybrid modeling approach incorporating structure prediction and validation, we show that ArlG filaments are open helices distinct to the closed helical filaments of ArlB. Curiously, further analysis reveals that ArlG retains a subset of the inter-protomer interactions of homologous ArlB, resulting in a superficially different assembly that nevertheless reflects the common ancestry of the two structures. This relatively simple mechanism to change quaternary structure was likely associated with the evolutionary neofunctionalization of the archaellar stator complex, and we speculate that the relative deformable elasticity of an open helix may facilitate elastic energy storage during the transmission of the discrete bursts of energy released by ATP hydrolysis to continuous archaellar rotation, allowing the inherent properties of a duplicated ArlB to be co-opted to fulfill a new role. Furthermore, agreement of diverse experimental evidence in our work supports recent claims to the power of new structure prediction techniques.

## Introduction

How does evolution produce novelty? At the molecular scale, evolution of new functions can appear difficult: How can a molecular machine with a dedicated function repurpose to perform a different function without passing through a less fit intermediate? Propulsive nanomachines, vital to movement of single-celled organisms toward favorable environments (Jarrell and McBride, 2008), demonstrate such repurposing with evolution of the novel function of cellular propulsion. Cilia, bacterial flagella, and archaella propel microbes by rotating, undulating, or whipping, yet evolved from non-propulsive ancestral machines (Beeby et al., 2020). Closer inspection indicates that redeployment of the mechanisms of pre-existing proteins for new roles has been a common theme in the evolution of these propulsive nanomachines. Such redeployment has been termed “exaptation,” distinguishing it from optimizing “adaptation” of a component’s mechanism for its current role (Gould and Vrba, 1982).

Archaella, the archaeal analogs of cilia or bacterial flagella, are rotary propellers that evolved from an ancestral non-rotary member of the type IV filament (TFF) family (Jarrell and Albers, 2012; Shahapure et al., 2014; Albers and Jarrell, 2018; Denise et al., 2019). Other members of the TFF family fulfill a variety of other roles: molecular grappling hooks that extend and retract their pili (type IVa pili), secretion systems with short pseudopili that extend and retract piston-like pumps (type II secretions systems), and surface adhesins that assemble non-retractile static filaments (including but not restricted to type IVb pili). All family members use a cytoplasmic ATPase to insert pilin subunits at the base of an extending polymeric filament, or pilus; some have additional paralogous ATPases that can also remove pilins to retract their filament. The directions, speeds, and forces with which pilins are inserted and removed form the mechanical basis for the function of different TFF family members. The archaellum is unique as a single ATPase allows for both assembly and rotation of the filament (Kinosita et al., 2016; Albers and Jarrell, 2018; Denise et al., 2019). How did the archaellum evolve to rotate without recruiting additional energy transducing proteins? Implicated in its evolution is that the TFF ATPases may intrinsically rotate, suggesting that their rotary mechanism was exapted to rotate the archaellar filament. To prevent exerted torque from futilely spinning the archaellar motor within the membrane, however, the archaellar motor must first be anchored (Kinosita et al., 2016; Beeby et al., 2020), enabling a filament-attached rotor component to rotate productively against a cell body-anchored stator component.

**FIGURE 1:**
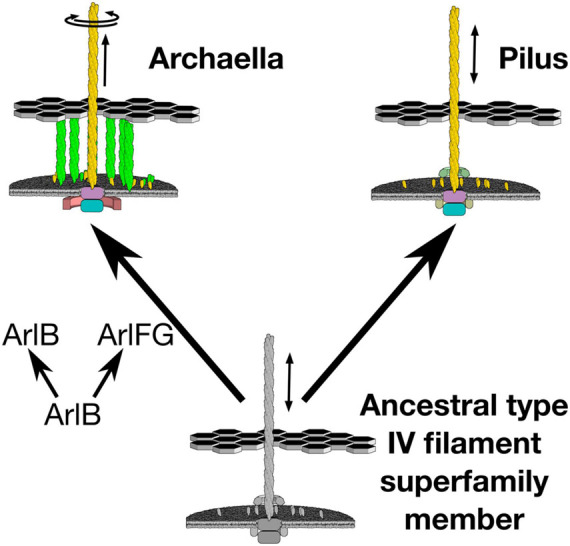
Evolution of archaellar rotary propeller function coincided with duplication of a member of the ArlB family. The archaellum uses a single ATPase to both extend and rotate its archaellar filament. Emergence of this new function coincided with duplication of the archaellar filament protein, ArlB, to also provide ArlF and ArlG, putative stator complexes against which the archaellar motor can push.

Indeed, recent results suggest that the key step in developing rotary motion may have been evolution of a stator that anchors to the cell ultrastructure, enabling the archaellar motor to act as a rotor that pushes against the stator to spin the extracellular filament (Figure 1). In the archaellum, the function of the stator complex may be fulfilled by ArlF and ArlG [note that archaellar proteins have recently been renamed “Arl” from “Fla” to clarify their unrelatedness to bacterial flagellar genes; ArlF and ArlG were previously referred to as FlaF and FlaG (Pohlschroder et al., 2018; Beeby et al., 2020)]. Both proteins feature an N-terminal transmembrane helix and a C-terminal soluble domain. *arlF* and *arlG* are conserved in the archaellar operon and are essential for archaellum filament formation and motility (Bayley and Jarrell, 1998). Studies in the model crenarchaeon *Sulfolobus acidocaldarius* revealed that both proteins are secreted into the pseudoperiplasm. Whereas full-length ArlF is secreted, ArlG has its N-terminus proteolytically cleaved upon secretion by an as-yet unidentified protease. *In vitro*, N-terminally truncated variants of ArlF (sArlF) and ArlG (sArlG) are stable at pH 3, making them stable in the acidic *S. acidocaldarius* pseudoperiplasm. ArlF interacts with the S-layer, the outer layer of the *S. acidocaldarius* cell envelope, and this interaction is required for motility. Additionally, the cleaved forms interact *in vitro* to form a heterotetramer; disrupting their binding interfaces abolishes motility (Bayley and Jarrell, 1998). Curiously, ArlF and ArlG share the same β-sandwich “archaellin” fold as ArlB, suggesting that one or both may polymerize to form a filament, although their N-terminal helices are 15–20 amino acids shorter. Indeed, our recent work found that sArlG forms a filament, while addition of ArlF interferes with ArlG filament formation (Banerjee et al., 2015; Tsai et al., 2020). Together, this suggests that ArlG forms a pseudoperiplasm-spanning filament with an S-layer-binding ArlF cap. The structural similarity of ArlF and ArlG indicates that the archaellar stator complex evolved from a duplication and neofunctionalization of a pre-existing component, the ancestral pilin itself (Ganfornina and Sánchez, 1999; Wang et al., 2019). What structural changes in the ancestral duplicated ArlB facilitated this neofunctionalization?

Understanding the structural changes required to form the first archaellar stator complex from an ancestral ArlG duplication would benefit from understanding its molecular structure. Although structures of neither the ArlG filament nor its ArlF cap exist, their crystal structures are known, and recent low-resolution imaging indicate that the quaternary structure of the ArlG filament differs from the ArlB filament: ArlB forms a compact helical filament where the hydrophobic interactions between the N-terminal helices form the tightly packed core of the archaellum filament, whereas 2-D projection images of ArlG filaments suggest an open helical structure (Daum et al., 2017; Tsai et al., 2020). Toward understanding the evolution of the archaellum from a non-rotary ancestor, here we describe the molecular architecture of the ArlG filament as inferred by a combination of cryomicroscopy data and a hybrid modeling approach incorporating structure prediction and validation.

## Results

### Preparation and cryoEM Imaging of ArlG Filaments

**FIGURE 2:**
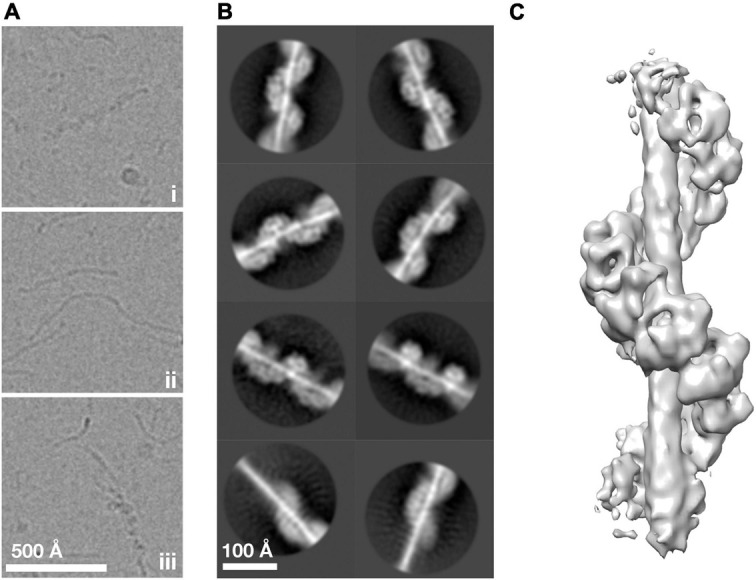
ArlG forms a helical filament. **(A)** Representative examples of purified *P. furiosus* sArlG demonstrating **(i)** helical filaments corresponding to previous results. Micrographs also featured **(ii)** thinner filamentous structures and **(iii)** helical filaments that transitioned to thinner filaments, mid-filament. **(B)** Representative 2-D classes on helical filaments; the bottom row shows 2-D classes of helical-to-thinner transitions, confirming their relation. **(C)** Isosurface of the final 3-D reconstruction of sArlG helical filament reveals an open helix of repeating densities around a central density.

To understand how ArlG neofunctionalized from an ancestral ArlB-like protein, we sought to understand the structural basis of ArlG oligomerization using single particle analysis cryoEM. Building on our previous work, we used the soluble N-terminally truncated *Pyrococcus furiosus* ArlG (sArlG) optimized for filament formation that mimics the physiologically relevant form of ArlG, which undergoes proteolytic cleavage upon secretion. We recombinantly overexpressed sArlG in *E. coli* and purified it using affinity and size exclusion chromatography as described previously (Tsai et al., 2020). We selected fractions from the first elution volume peak for further analysis because they produced the longest sArlG oligomers based on electron microscopy of negatively stained sample. We vitrified sArlG filament suspensions on holey grids and acquired micrographs using a Titan Krios with a Falcon III direct electron detector camera. Micrographs revealed filaments approximately 100 Å thick that suggested helical structures (Figure 2Ai), comparable to previous negative stain results (Tsai et al., 2020). Despite our sample lacking significant contamination (Supplementary Figure 1), our micrographs also featured thin filaments (Figure 2Aii). In some cases, we also saw thick filaments transitioning into thin filaments (Figure 2Aiii). It is unclear what the thin or transitional filaments represent, with possibilities including alternative denatured structures at the air-water interface or cleavage (despite not appearing our gels).

### ArlG Forms a Helical Filament

We used single particle analysis cryoEM to determine the structure of the sArlG filament. We used crYOLO (Wagner et al., 2019) to automatically pick 323,770 particles from micrographs at 30 Å intervals and RELION (Zivanov et al., 2018) to produce 2-D classes representing different views of the sArlG filament (Figure 2B). In all 2-D classes we observed a helix comparable to our previous results, wrapping around a central density not evident in earlier negative stain reconstructions; some classes captured the full filament transitioning to the thinner filamentous density (Figure 2B, bottom), reinforcing that this central density is synonymous with the thin filaments observed in our raw micrographs.

Repeated attempts at helical reconstruction using RELION failed to produce 3-D classes with distinguishable features; instead, we treated particles as single particles for 3-D classification, as has been successfully used by other groups (Shibata et al., 2019). This approach yielded a best 3-D class containing 40,224 particles. After transitioning to cryoSPARC, 3-D refinement and post-processing yielded a map with a reported resolution of 9.1 Å Gold standard FSC (Figure 2C and Supplementary Figure 2). This map revealed a helix of repeating hollow subunits; that these subunits were repeating structures validated our approach as no symmetry was applied during reconstruction. We could not determine the handedness at this stage without *a priori* information. To assess the influence of the thin central density, which lacked clear features in our reconstruction, on alignment, we attempted signal subtraction of this density (Bai et al., 2015). Signal subtraction resulted in a structure comparable to that of the non-subtracted density maps, indicating that the central filamentous density does not significantly affect alignment or reconstruction. A turn of the helix in our final structure had 7.8 protomers, a radius of 44.4 Å, and a twist of −46.1° and rise of 14.7 Å per subunit.

### Predicted and cryoEM Structures Independently Agree on the Molecular Structure of the ArlG Helix

Because the resolution of our map was insufficient for *de novo* model building, we used a hybrid approach to build and validate a molecular model of the ArlG helix. We first assessed how sArlG monomers may assemble to form a repeating helical filament by evaluating possible orientations of the only known crystal structure of ArlG, that of *S. acidocaldarius*, into our map. As absolute handedness cannot be determined from 2-D projection images (Sorzano et al., 2006; Zivanov et al., 2018), monomeric sArlG was fitted into maps of both handedness using SegFit in UCSF Chimera (Pintilie et al., 2010). The monomer fitted better in the left-handed map, and we independently identified similar best fits of the monomer in two of the repeating subunit densities.

**FIGURE 3:**
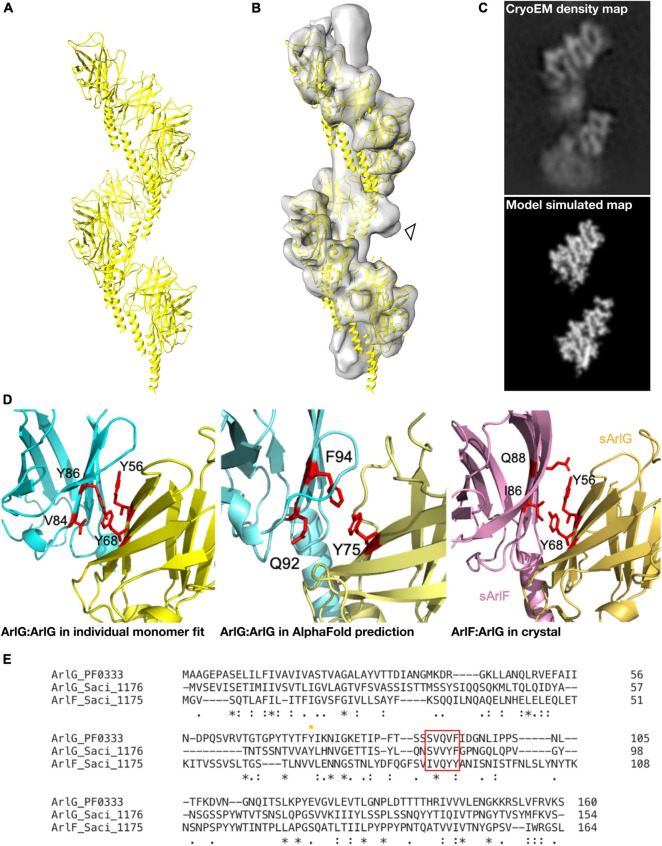
A molecular model of the ArlG helical filament is supported by diverse observations. **(A)** Molecular model of a helical filament ArlG from AlphaFold 2. **(B)** Demonstration of fit of molecular model within our cryoEM density map. Arrowhead highlights unexplained non-regular density **(C)** Validation of our molecular model by comparison of density map of our cryoEM structure (top) and simulated model density map (bottom). Note that small differences in helical parameters lead to loss of register in the lower sections of the two maps. **(D)** Illustration of consistent interfaces between monomers in (left) interaction surface of two sArlG monomers (PDB 5TUH) independently fitted into two subsequent repeats in our cryoEM density map, (middle) interface between two subsequent protomers in the *de novo* AlphaFold model, and (right) ArlG interface with paralogous ArlF in previously determined crystal structure (Tsai et al., 2020). **(E)** Multiple sequence alignment of *P. furiosus* ArlG and *S. acidocaldarius* ArlG and ArlF performed with Clustal Omega highlighting conserved interface residues. The key ArlG tyrosine residue (Y68 in *S. acidocaldarius*, Y75 in *P. furiosus*) at the ArlG-ArlF interaction site is indicated with a yellow asterisk, and residues in ArlG corresponding to the residues of ArlF at the interaction site are highlighted by the red box.

In parallel, we used AlphaFold 2 (Jumper et al., 2021) to model the structure of an ArlG oligomer *de novo* without any *a priori* information on our cryoEM structure or the fold of ArlG. We predicted the structure of a monomer, pentamer, octamer and decamer of ArlG using the sequence of our *P. furiosus* sArlG. The monomeric structure accurately predicted the crystal structure of sArlG (RMSD 4.7 Å), while the pentamer, octamer, and decamer all formed comparable left-handed open helices. We extrapolated these helices by iteratively superimposing overlapping subcomplexes to form a 13-mer, and inspected this to determine that it forms a left-handed helix with a helical turn consisting of 8.1 protomers, a helical radius of 41.7 Å, and a twist of −44.4° and rise of 12.8 Å per subunit, a close match with our cryoEM density map (Figure 3A). We were unable to compare AlphaFold 2 results to the analogous RoseTTaFold because it does not currently offer homooligomer structure prediction (Baek et al., 2021).

Four independent observations supported the validity of this molecular model. First, our helical model fitted well into the cryoEM density map (Figure 3B) and a simulated low-pass filtered density map from our molecular model corresponded to the observed densities in our cryoEM map (Figure 3C). Second, the residues at the interaction site between *P. furiosus* ArlG monomers in our model corresponded to residues forming the interface between ArlG monomers when independently fitting two *S. acidocaldarius* sArlG crystal monomers into our cryoEM density map: Y75 in one monomer, and Q92 and F94 from the other monomer, corresponding to Y68, V84 and F86, respectively, in *S. acidocaldarius* (Figure 3D). Third, the interaction site between ArlG monomers in our model corresponded to residues involved in crystal packing between *S. acidocaldarius* ArlG and its paralog ArlF: Y56 and Y68 from one monomer, and V84 and Y86 from the other monomer (Figures 3D,E; Tsai et al., 2020). Fourth, we later discovered (see below) that a subset of the inter-protomer interfaces in the filament formed by distant paralog ArlB closely match the binding interfaces of ArlG; ArlB residues Y77 and V95 are structurally equivalent to ArlG residues Y75 and Q92, respectively, (Supplementary Figure 4). We consider the likelihood of such a coincidence between two homologous proteins occurring by chance as small.

### The Distinct Quaternary Structure of ArlG Can Be Explained by Retention of a Subset of the Oligomerization Surfaces of ArlB

**FIGURE 4:**
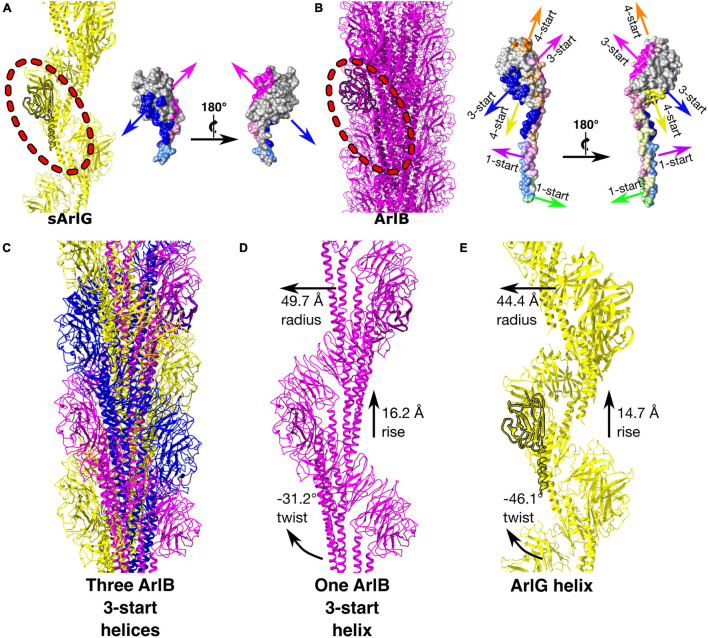
sArlG forms a helical filament with a different quaternary structure to the ArlB filament yet using a subset of the interactions used by ArlB. Comparison of the quaternary structures of the *P. furiosus* sArlG and ArlB filaments. **(A)** Left: Model of the sArlG filament (yellow) with a single protomer highlighted (outline). Right: two views of intersubunit sArlG interactions mapped onto the surface of a single protomer; blue represents interactions with the preceding protomer while pink represents interactions with the next protomer. Full colors represent interactions to the other subunit’s archaellin domain while pastels represent interactions to the N-terminal α-helix tail. **(B)** Left: Model of the ArlB filament from PDB 5O4U (purple) with a single protomer highlighted (outline). Six colors represent preceding and next protomers in the 1-start, 3-start, and 4-start helices of the filament. The 3-start helix interactions of ArlB (blue and pink) correspond to the intersubunit interactions of sArlG. **(C)** Illustration of the three 3-start ArlB helices in yellow, magenta, and blue. **(D)** The interactions and helical parameters of a single extracted 3-start helix of ArlB resembles **(E)** the sArlG helix.

Comparison of the sArlG and ArlB filament structures demonstrated different quaternary structures (Figure 4). sArlG forms an open helical filament of soluble archaellin domains, with each subunit contacting only the previous and next subunits (Figure 4A). The tight closed helical structure of the ArlB filament, on the other hand, consists of a hydrophobic core of its N-terminal α-helical domain, with the soluble C-terminal archaellin domains lining the outside of the filament (Daum et al., 2017; Figure 4B). Each *P. furiosus* ArlB archaellin domain contacts six other subunits, resulting in 1-start, 3-start, and 4-start helices.

Strikingly, the interaction surfaces used by ArlB in the 3-start helix closely resemble those used by ArlG in its interactions with neighboring protomers (Figure 3D and Supplementary Figure 4). Upon inspection of the three 3-start ArlB helices (Figure 4C), we noted that a single 3-start helix resembles the structure of our sArlG filament: a turn of this speculative extracted helix has 11.6 protomers per turn, a radius of 49.7 Å, and a twist of −31.2° and rise of 16.2 Å per subunit (Figures 4D,E).

## Discussion

Because the ArlFG putative stator complex is exclusive to the archaellar motor and no other members of the TFF superfamily, understanding its origin is key to understanding the origin of archaellum rotation (Denise et al., 2019; Beeby et al., 2020). ArlG and ArlF are products of duplication and neofunctionalization of the filament-forming archaellin protein ArlB (Banerjee et al., 2015; Denise et al., 2019; Wang et al., 2019; Tsai et al., 2020). This neofunctionalization resulted in an ArlF-capped ArlG filament that bridges the pseudo-periplasm to anchor to the S-layer (Tsai et al., 2020). Understanding the changes of these proteins that resulted in neofunctionalization from a propeller to a stator thus provides a specific opportunity to study how molecular machines evolve by “tinkering” of existing components (Beeby et al., 2020). Here we present the first molecular model of the ArlG filament, allowing insights into the role of a gene duplication in the evolution of a rotary motor.

Our structure supports previous findings that ArlG filaments have a distinct quaternary structure to ArlB filaments (Tsai et al., 2020; Figures 3, 4). Curiously, however, the differences between the ArlB and ArlG filaments are not the result of evolution of an entirely new set of interaction surfaces. Rather, more subtle shifts have occurred in the divergence of ArlB and ArlG. In ArlB, filament assembly is driven by hydrophobic packing of the long N-terminal α-helix of each monomer together with inter-archaellin domain interactions (Daum et al., 2017), while the ArlG filament assembles primarily around stacking interactions between archaellin domains (Figure 4). Nevertheless, the ArlG filament still uses interactions from its shorter N-terminal α-helix, and its stacking interactions with two other ArlG protomers is a subset of the six interactions seen between ArlB protomers.

How the shift in interactions leading to these quaternary structure differences evolved is not clear. Because the ArlB filament extensively uses interactions around a hydrophobic core formed by its N-terminal α-helix, mutations in the soluble archaellin domain, less important for filament formation, may therefore have been tolerated. After gene duplication, one paralog would be relieved of functional constraints, allowing neutral drift to facilitate the loss of four of the inter-archaellin domain interactions, and loss of interactions that hold together the 1-start and 4-start helices. Consolidation of the 3-start interactions might also lead to less reliance upon the N-terminal α-helical hydrophobic core, which could subsequently shrink (Daum et al., 2017). Such a pattern of evolution has been referred to as elimination of functional redundancy (Thornhill and Ussery, 2000), and offers a straightforward explanation of the apparently irreducible complexity of evolution of a new subcomponent. Why the ArlG filament features a single helix instead of a triplet of loosely associated helices, resembling the three 3-start ArlB helices, is not immediately obvious, but may stem from steric clashes preventing assembly of intertwined helices. Indeed, we observed sporadic additional blobs that project from the central density between the open helical band of ArlG in our cryoEM maps that may be the residual of failed assemblies of additional intertwined ArlG helices (see arrowhead, Figure 3B).

Our study also brings some unexpected secondary findings. The nature of the thin central density remains a mystery, although it is also apparent along the center of the ArlG open helix. Because our protein preparations lack significant contaminants, the most parsimonious explanation is that this density is also ArlG. Whether it is an alternate conformation, a degradation product, or something else altogether remains to be answered. Comparison to the extracted ArlB 3-start helix suggests that this density may result from a bundle of α-helical helices, but we cannot explain why we also see it alone without a helix of C-terminal ArlG archaellin domains.

We cannot rule out that filament formation by ArlG is an *in vitro* artifact of a truncated, *ex situ* protein. After all, such a helical structure has never been observed in low-resolution *in situ* electron cryotomography structures of the archaellum (Briegel et al., 2017; Daum et al., 2017), filaments are relatively easy oligomeric states to assemble at random (Egelman, 2003), and our modest resolution prevented us from unambiguously identifying ArlG within the density. Nevertheless, on balance we believe our results are biologically relevant. Independent fitting of the crystal structure of two sArlG monomers gave comparable orientations for each monomer (Figure 3). The resulting predicted interfaces are supported by sequence conservation and are independently corroborated by crystal packing between ArlG and its ArlF homolog, and AlphaFold predicted quaternary structure. That the ArlG helix is not seen in electron cryotomography studies is perhaps less concerning when one considers that the analogous bacterial flagellar stator complexes have never been seen in model organisms such as *Salmonella enterica* (Beeby et al., 2016), despite considerably higher data quality; archaellar stator complexes may be present unevenly distributed around the structure and therefore be invisible after subtomogram averaging. We are also confident that our helical structure is not an imaging artifact: by taking a single particle analysis approach and applying no symmetry during reconstruction, the repeating motifs of density cannot be artifacts of applied symmetry. They also nicely match our simulated density from our Alphafold structure prediction of a pentameric homo-oligomer that forms repeating units of a similar, helical nature. That our ArlG helical filaments are reproducible between studies lends further confidence to our work (Tsai et al., 2020).

Other key questions remain that could guide future work. A crucial next step will be to improve the resolution of our structure so we can test our current molecular model and identify the central density *de novo.* The ArlG filament is likely flexible due to the relative paucity of inter-protomer interactions compared to ArlB. Indeed, our 2-D class averages reveal curvature in some classes (Figure 2B), suggesting that approaches to stabilize the structure of the helix may facilitate improved datasets. Interaction studies will be vital to identify how the ArlG filament interacts with the archaellar motor, although this may be challenging because these interactions may be the rotation interface (Tsai et al., 2020). Furthermore, reconstitution and imaging of ArlF-capped ArlG filaments, and higher resolution *in situ* imaging of the stator complex in cells–or with purified S-layer fragments–is needed to confirm that this putative stator complex is physiological.

Our findings support the putative function of ArlG as a stator complex protein that forms a left-handed filament that spans the pseudoperiplasm to bind the S-layer via ArlF (Figures 2C, 3). The energized rotor component of the archaellum can push against this anchored stator complex for productive rotation of the archaellar filament (Banerjee et al., 2015; Tsai et al., 2020). Because the predicted ArlG:ArlG interface echoes the ArlG:ArlF interface in their crystal structure, ArlF may cap the assembling ArlG filament when in the presence of an S-layer scaffolding to position ArlF (Tsai et al., 2020). These findings also support that quaternary structure changes were involved in the neofunctionalization to form the stator complex following gene duplication of ArlB (Figure 4) through loss of interaction surfaces.

It has not escaped our notice that the specific structure of an open helix for the stator complex immediately suggests a possible elastic storage mechanism for transmission of the relatively large packets of energy released by ATP hydrolysis to continuous rotation of the archaellar filament, smoothing the transitions from one step to another (Iwata et al., 2019).

## Materials and Methods

### Data Availability

Our sArlG construct has been previously described in Tsai et al. (2020) and is available on request. The sArlG Coulomb potential map has been deposited in the Electron Microscopy Data Bank (EMDB) (accession number EMD-13428). The atomic coordinates of our sArlG 13-mer model can be downloaded from http://www.beebylab.org/downloads/ or on request from MB.

### Protein Expression, Purification, and cryo-EM Sample Preparation

Bacterial strains, protein expression and purification are detailed in Tsai et al. (2020). Size exclusion chromatography fractions 11–13 mL were pooled and 3 μL applied to plasma cleaned for 60 s in air mixture, negatively charged Quantifoil R2/2 grids. Grids were plunge frozen using an FEI Vitrobot Mark IV using 3 s blot time, –3 blot force, 95% humidity and screened on a Tecnai F20 with Falcon II DED.

### Data Acquisition

Micrographs of purified *P. furiosus* sArlG filaments were obtained by electron cryomicroscopy. Collection parameters detailed in Table 1.

**TABLE 1:** Collection of electron cryomicroscopy images of purified sArlG filaments.

**Data collection**	
Electron microscope	Titan Krios G2
Electron detector	Falcon III DE
Voltage (keV)	300
Magnification	96,000 × (nanoProbe)
Pixel size (Å)	0.85
Defocus range (μm)	–1.5 to –2.9
Defocus step (μm)	0.3
Dose rate (e^–^Å^–2^s^–1^)	0.86
Integration time (s)	40
Number of raw movies	4632
Number of frames per movie	32

### Image Processing Using RELION, crYOLO, and cryoSPARC

Data was processed using RELION-3.1.1 (Scheres, 2012; Zivanov et al., 2018). Raw movies were pre-processed by motion correction using RELION’s MotionCor2 (using all frames) (Zheng et al., 2017), and CTF estimation with CTFFIND4 (Rohou and Grigorieff, 2015). Micrographs with low figure of merit scores (< 0.0275), low contrast, poor defocus estimates or large amounts of unwanted ice were removed.

The neural network-based particle picking software SPHIRE-crYOLO was trained using 302 manually picked sArlG filaments from 39 micrographs and picked 49,271 sArlG filaments from all micrographs (filament width 120 pixels, box overlap 24 pixels) (Wagner et al., 2019, 2020). Filament coordinates were imported into RELION-3.1.1; 323,770 particles were extracted (box size 300 pixels) and rescaled by half.

Preliminary structural analysis revealed helical filaments, but a single particle analysis approach yielded superior results to helical processing (He and Scheres, 2017; Tsai et al., 2020). Particles picked by SPHIRE-crYOLO were processed by 2-D and 3-D classification (spherical mask 200 Å). Particles within 5 Å of each other after alignment were removed from the best 3-D class to mitigate map over-fitting leaving 40,224 particles. and the non-duplicated particles put through 3-D refinement (Scheres and Chen, 2012; Zivanov et al., 2018). A mask was created from the refined 3-D map (extended by 0 pixels, 10-pixel raised-cosine soft edge) for post-processing, which estimated a B-factor of –59.9.

Complementary image processing was performed in cryoSPARC 3.2 (Punjani et al., 2017) using particles imported from prior RELION processing. Particles were input to the homogeneous refinement workflow and then subsequent map sharpening was performed with –427.6 Bfactor applied.

### Modeling the Structure of the sArlG Filament With AlphaFold 2.0

Structure prediction using Alphafold 2 was performed using Google Colab notebooks (Mirdita et al., 2021) using a cloud-based runtime session utilizing an NVIDIA Tesla T4 GPU. Multiple sequence alignment was performed against the sequence of *Pyrococcus furiosus* sArlG using jackhmmer and all other parameters were kept as default in the Advanced notebook. Homooligomers of 5, 8, and 10 subunits of sArlG were predicted with 5 different model parameters and the models that fit our maps best were used for analysis.

### Estimation of Helical Parameters

Helical parameters were determined from volume maps produced during cryoEM reconstruction. For comparable estimates of helical parameters, PDB coordinates from Alphafold 2.0 were converted to MRC volumes using e2pdb2mrc.py from the EMAN2 package (Tang et al., 2007). Volumes were simulated using identical MRC header characteristics, simulated resolution of 8 Å. Initial estimates of helical parameters were measured by first exporting images of flattened volumes in different orientations from 3dmod Slicer (IMOD package) (Kremer et al., 1996). These images were imported into ImageJ, distances and angles were manually measured (Schneider et al., 2012). Manually estimated parameters were then used to inform search ranges in cryoSPARC’s symmetry search utility, from which the result with the lowest mean squared error was chosen.

### Model Visualization and Comparison With ArlB Filament

3-D models were assessed by visualizing the isosurface in UCSF Chimera (Pettersen et al., 2004) and volume slices in 3dmod Slicer from the IMOD package (Kremer et al., 1996). The sArlG monomer crystal structure (PDB: 5TUH) was fitted into the filament model with SegFit in UCSF Chimera (Pettersen et al., 2004; Pintilie et al., 2010). Absolute handedness cannot be directly determined from 2-D projection images such as micrographs (Sorzano et al., 2006; Zivanov et al., 2018), thus the sArlG filament map was flipped using the RELION image handler (Scheres, 2012) and monomer fitted into left- and right-handed models. Molecular models of the ArlG and ArlB (PDB: 5O4U) filament structures were visualized with UCSF ChimeraX (Pettersen et al., 2021) and residues at interaction interfaces were visualized in PyMol (Schrödinger, 2015). Sequence alignment of *P. furiosus* ArlG and ArlF was performed using Clustal Omega (Madeira et al., 2019).

## Data Availability Statement

The datasets presented in this study can be found in online repositories. The names of the repository/repositories and accession number(s) can be found below: https://www.ebi.ac.uk/pdbe/emdb/, EMD-13428.

## Author Contributions

MB and S-VA conceived and designed the study. TU, JB, and SS purified proteins and prepared samples for imaging. TU coordinated data acquisition. TU and YW processed and analyzed data including structural modeling and analysis. MB, TU, and YW interpreted findings and wrote the manuscript. All authors contributed to manuscript revision, read, and approved the submitted version.

## Conflict of Interest

The authors declare that the research was conducted in the absence of any commercial or financial relationships that could be construed as a potential conflict of interest.

## Publisher’s Note

All claims expressed in this article are solely those of the authors and do not necessarily represent those of their affiliated organizations, or those of the publisher, the editors and the reviewers. Any product that may be evaluated in this article, or claim that may be made by its manufacturer, is not guaranteed or endorsed by the publisher.
