# Supplementary material for: Evolution of Archaellum Rotation Involved Invention of a Stator Complex by Duplicating and Modifying a Core Component
Source: Front Microbiol. 2021 Nov 29;12:773386. doi: 10.3389/fmicb.2021.773386 (PMC8667602; doi:10.3389/fmicb.2021.773386)
Supplement: Supplementary file 1 [file Data_Sheet_1.docx]

# Supplementary Figures and Data

| *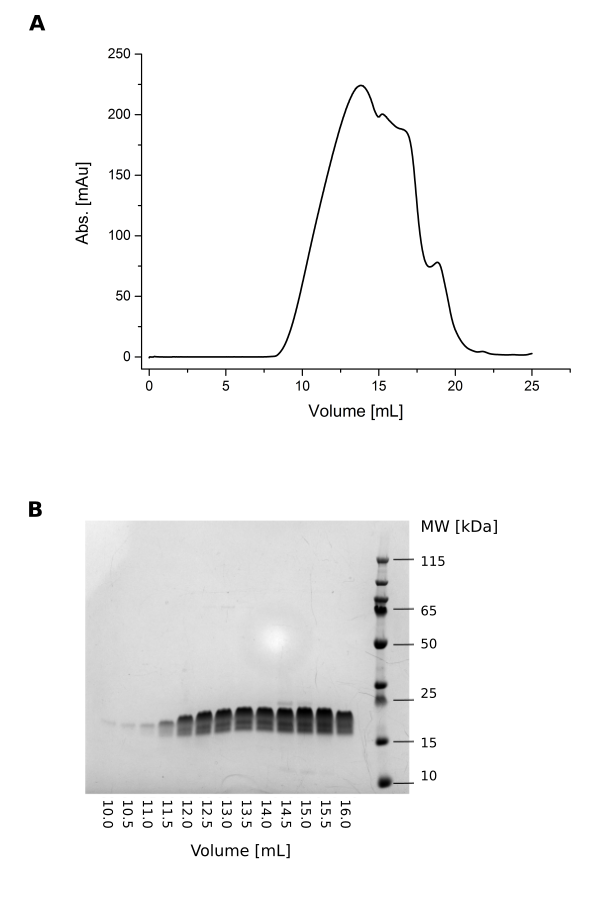* |
| --- |
| **Supplementary Fig. S1: Recombinant expression and purification of sArlG. (A)** Size exclusion chromatography trace from purification of recombinant sArlG. **(B)** SDS-PAGE of the SEC peak. |

| *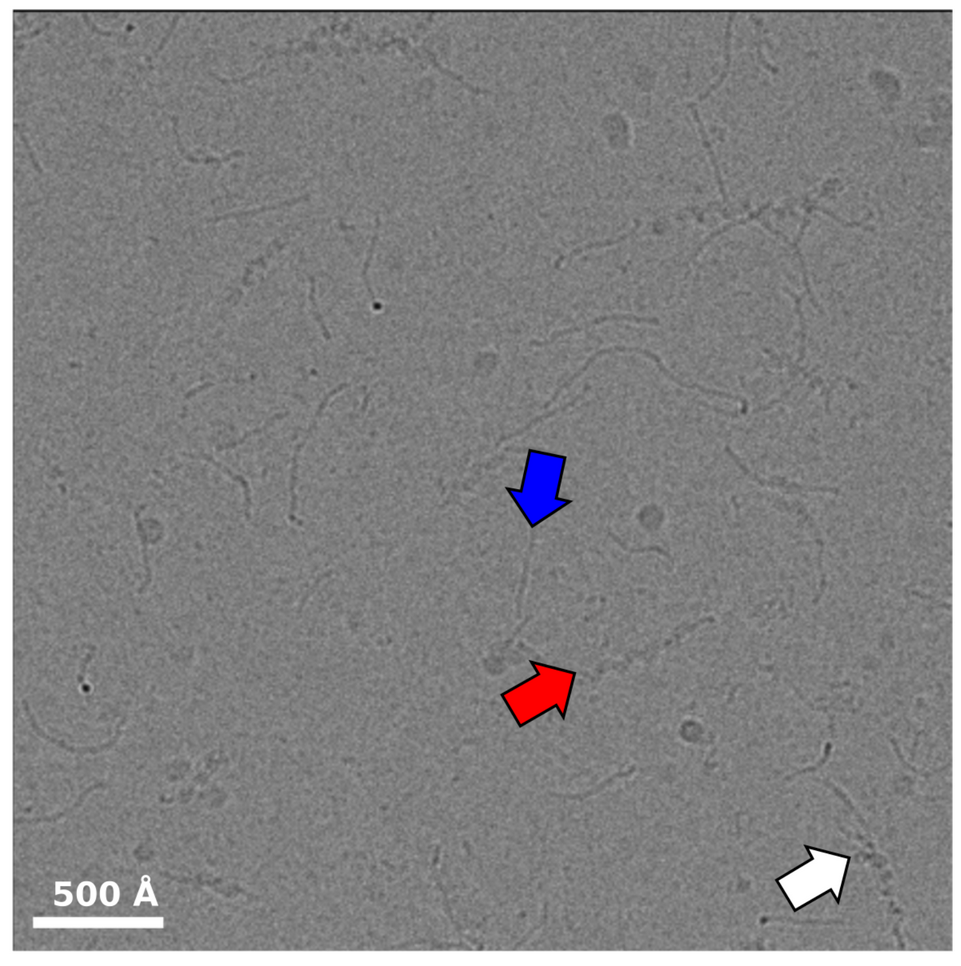* |
| --- |
| **Supplementary Fig. S2:** Representative micrograph of purified *P. furiosus* sArlG demonstrating helical filaments corresponding to previous results (red arrow). Micrographs also featured thinner filamentous structures (blue arrow), and helical filaments that transitioned to thinner filaments, mid-filament (white arrow). Arrows point to features in Figure 2A(i-iii). Bar, 50 nm. |

| *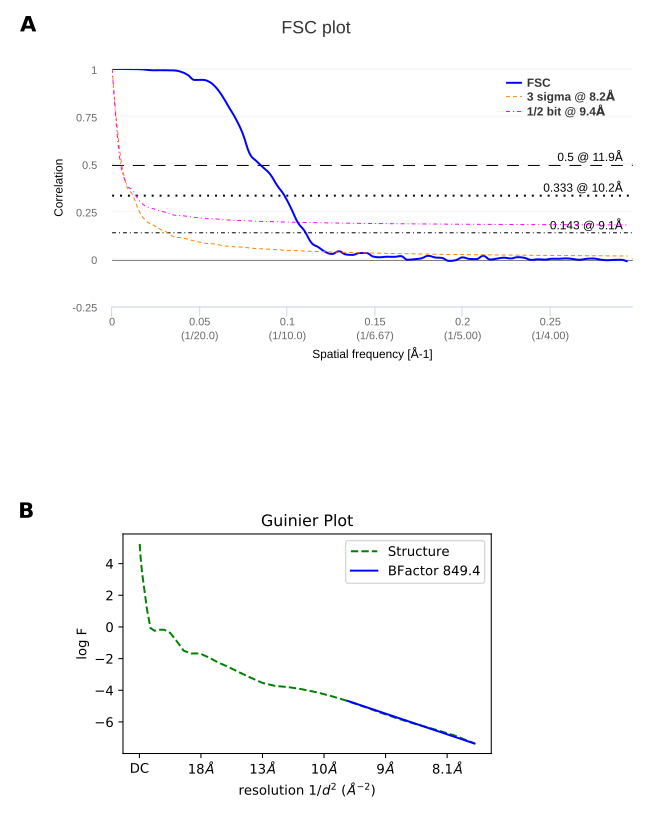* |
| --- |
| **Supplementary Fig. S3: sArlG reconstruction details. (A)** Fourier Shell Correlation (FSC) curves calculated between two independently refined half-maps. Gold standard FSC (0.143) determined 9.1 Å resolution. Calculated using EMDB FSC server. **(B)** Guinier Plot output from cryoSPARC 3.2, Bfactor 849.4. |

| *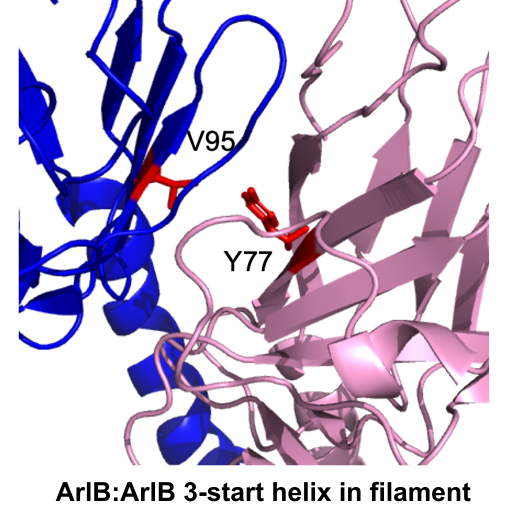* |
| --- |
| **Supplementary Fig. S4: ArlB:ArlB 3-start helix interfaces in the archaellum filament.** Illustration of the 3-start helix interface between monomers of ArlB (PDB 5O4U) in the archaellum filament, paralogous and consistent with the ArlG:ArlG and ArlF:G contact interfaces in Figure 3D. |

# 
